# Cerebral Hemispheric Lateralization Associated with Hippocampal Sclerosis May Affect Interictal Cardiovascular Autonomic Functions in Temporal Lobe Epilepsy

**DOI:** 10.1155/2016/7417540

**Published:** 2016-02-24

**Authors:** Rokia Ghchime, Halima Benjelloun, Hajar Kiai, Halima Belaidi, Fatiha Lahjouji, Reda Ouazzani

**Affiliations:** ^1^Physiology Laboratory, Faculty of Medicine and Pharmacy, Mohammed V-Souissi University, 6203 Rabat, Morocco; ^2^Department of Clinical Neurophysiology, Hospital of Specialties, Ibn Sina University Hospital, Rabat Institute, 6220 Rabat, Morocco; ^3^Unit of Cardiology A, Ibn Sina University Hospital, 10000 Rabat, Morocco; ^4^Food Sciences Laboratory, Department of Biology, Faculty of Sciences Semlalia, Prince Moulay Abdellah Avenue, 40090 Marrakesh, Morocco

## Abstract

It is well established that the temporal lobe epilepsy (TLE) is linked to the autonomic nervous system dysfunctions. Seizures alter the function of different systems such as the respiratory, cardiovascular, gastrointestinal, and urogenital systems. The aim of this work was to evaluate the possible factors which may be involved in interictal cardiovascular autonomic function in temporal lobe epilepsy with complex partial seizures, and with particular attention to hippocampal sclerosis. The study was conducted in 30 patients with intractable temporal lobe epilepsy (19 with left hippocampal sclerosis, 11 with right hippocampal sclerosis). All subjects underwent four tests of cardiac autonomic function: heart rate changes in response to deep breathing, heart rate, and blood pressure variations throughout resting activity and during hand grip, mental stress, and orthostatic tests. Our results show that the right cerebral hemisphere predominantly modulates sympathetic activity, while the left cerebral hemisphere mainly modulates parasympathetic activity, which mediated tachycardia and excessive bradycardia counterregulation, both of which might be involved as a mechanism of sudden unexpected death in epilepsy patients (SUDEP).

## 1. Introduction

Temporal lobe epilepsy (TLE) is well known to be associated with autonomic nervous system dysfunctions [1, 2]. Seizures alter the function of different systems such as the respiratory, gastrointestinal, urogenital, and, most importantly, cardiovascular system [1, 3, 4].

Autonomic cardiovascular functions are mainly regulated by cortical, midbrain, and brainstem areas [5, 6]. The vital cortical area is the insular cortex folding into the inner temporal lobe that is often dysfunctional in TLE. Lesions of the insular cortex are usually associated with abnormalities in heart rate (HR) and blood pressure (BP) regulation as well as with cardiac arrhythmia [7]. In addition, mesiotemporal structures were reported to be involved in both epileptogenesis and autonomic control in animal models [8]. Besides, several studies also indicate that the insular cortex and temporal lobe are powerful in the control of cardiovascular function in humans [7–10].

Hemispheric centers of autonomic nervous control show distinct modulation activities. For instance, while the right hemisphere modulates the sympathetic cardiovascular activity, the left hemisphere contributes more to the parasympathetic activity. These modulation discrepancies occur in various neurologic disorders, such as intracranial tumors, cerebral trauma, encephalitis, hemorrhagic or ischemic stroke, or epilepsy. In epilepsy patients, signs of autonomic dysfunction occur both interictally and ictally. During seizures, cardiovascular disturbances such as tachy- or bradyarrhythmia are frequent findings, indicating close interactions between epileptogenic brain areas and centers of autonomic control [11–13]. Supraventricular bradycardia has been highly reported in TLE with left HS compared to TLE with right HS and the tachycardia more reported in TLE with right HS compared to TLE with left HS, suggesting an increase of sympathetic cardiac activity caused by the right hemisphere innervations.

On that focus, our study is designed to compare the changes of heart rate (HR) and blood pressure (BP) in two groups of TLE, with right HS and with left HS to investigate the possible factors related to the structural characteristics of TLE, especially cerebral lateralization associated with hippocampal sclerosis.

## 2. Patients and Methods

### 2.1. Patients

This study was conducted between 2012 and 2015 at the Department of Clinical Neurophysiology and at the unit of autonomic nervous system (ANS) exploration at the Department of Cardiology A, University Hospital Center (UHC) Ibn Sina, Rabat, Morocco. It was approved by the Ibn Sina ethical committee. A written consent form was obtained from each patient before the tests.

Thirty patients with refractory temporal lobe epilepsy destined to surgery were recruited at the Department of Clinical Neurophysiology for pretreatment and diagnosis protocol, including clinical, neurophysiological, and electrophysiological examinations (video-EEG) as well as MRI and battery of tests for the assessment of cardiac autonomic functions. The study subjects have mesial temporal lobe epilepsy with complex partial seizures according to the classifications of the International League Against Epilepsy, 1981 and 1989. However, patients with lesions other than hippocampal sclerosis on cranial MRI; patients with EEGs within the last 24 hours showing interictal epileptiform discharges; subjects suffering from diseases other than epilepsy that are known to affect the autonomic cardiovascular system (renal failure, diabetes mellitus, and cardiopulmonary disease) were excluded. The following subjects were also expelled from our study: subjects diagnosed for psychiatric illnesses, or those with abuse of alcohol or smoking as well as patients breast-feeding, or in pregnancy [14, 15].

### 2.2. Methods

All patients underwent an autonomic cardiac function testing which is based on HR and BP responses at rest and after different stimuli. All tests were conducted under standardized conditions. The measurements were carried out in quiet room with an ambient temperature of 22°C between 9 and 12 a.m. Patients were asked to avoid taking any drugs other than their AEDs a week before the tests [16, 17]. The interval between the different tests was standardized so that the following test did not start until the HR and BP had returned to the baseline level after the preceding test. HR and BP responses at rest and after stimulation were recorded under the subsequent conditions: normal breathing, deep breathing, isometric work (hand grip), mental stress, and tilting. ECG was first recorded through the normal breathing. Consecutive RR intervals were measured from the ECG for a period of 5 min and the standard deviation of the intervals was employed as the test variable.

#### 2.2.1. Deep Breathing Test

This test evaluates the autonomic function by measuring the changes of HR in response to controlled breathing [16, 18]. During the test of deep breathing (DB), patients were asked to breathe deeply at frequency of six breaths per minute. The respiratory frequency has an influence on the variation of RR interval on the EKG. The vagal response to the DB is calculated using the following equation:

(1) $$\begin{array}{c} DB=\frac{\left({{RR}_{maximal}-RR}_{minimal}\right)}{{RR}_{minimal}}\times 100. \end{array}$$

RR_minimal_ and RR_maximal_ are intervals obtained at the end of expiration and the end of inspiration, respectively.

#### 2.2.2. Isometric Contraction or Hand Grip Test

During 3 minutes the subject carries out a manual pressure of 50% of the maximum using a dynamometer (Jetter and Scheerer, Germany) with his dominant hand and the maximum voluntary contraction (MVC) was calculated. BP was measured before and after contraction at 1, 2, and 4 min in the contralateral arm.

The muscular contraction involves an increase in BP related to a rise of sympathetic nervous system at the muscular level that is effort- and time-dependent [19, 20]. The peripheral sympathetic nervous response “*α*” is given by the increase of the BP:

(2) Peripheral  sympathetic  response  “α”=BPafter  the  test−BPbefore  the  testBPbefore  the  test×100.

#### 2.2.3. Mental Stress Test

The patient performs mental arithmetic calculations by removing the number 7 successively from 200. The result is an augmentation in HR and in BP by activation of the central sympathetic nervous system [21].

In mental stress, the central sympathetic nerves activities “*α*” were assessed by measuring the changes of BP as follows [19, 20]:

(3) Central  sympathetic  response  “α”=BPafter  stimulation−BPbefore  stimulationBPbefore  stimulation×100.

The central sympathetic nervous activity “*β*” was assessed by measuring the changes of HR as follows [19, 20]:

(4) Central  sympathetic  response  “β”=HRafter  stimulation−HRbefore  stimulationHRbefore  stimulation×100.

#### 2.2.4. Orthostatic or Tilt Test

The responses of HR and BP to quick postural change (from standing to supine position) were recorded. Systolic and diastolic BP were measured at rest and immediately after and during standing position. The difference between BP at rest and the lowest BP after standing was calculated [14, 22]. The assessment of peripheral sympathetic response beta is given by the following formula:

(5) Peripheral  sympathetic  response  “β”=BPafter  stimulation−BPbefore  stimulationBPbefore  stimulation×100.

In both orthostatic and mental stress tests, values above 10% correspond to sympathetic hyperactivity; a response equal to 10% is considered normal. However, patients with values below 10% are deficient.

### 2.3. MR Imaging

Patients underwent a standard MRI protocol that was carried out using a 1.5-T scanner (Philips Intera System). Imaging studies included (1) FLAIR, T1 IR, and T2 weighted hippocampal images, coronal oblique images perpendicular to the long axis of the hippocampus, with a 2 mm slice thickness and no intervening gap and (2) axial T1 and T2 weighted, coronal T2 weighted, and sagittal T1 weighted cranial images with a 5-mm slice thickness and 1.5-mm intervening gap [17, 18, 20].

### 2.4. EEG

Interictal EEG was recorded several times before the autonomic cardiac function testing, employing a video-EEG monitoring system (Micromed; Treviso, Italy); the electrodes were arranged according to the International 10-20 system. The duration of video-EEG monitoring varied from 2 to 7 days, and at least three habitual seizures were recorded for each patient.

## 3. Statistics

Data were analyzed using the SPSS software (version 15.0; SPSS Inc., Chicago, IL, USA). The tests selected for the comparisons between the two studied groups, TLE with right hippocampal sclerosis and TLE with left hippocampal sclerosis, were the following: mental stress for the study of alpha and beta central sympathetic nerve activities; the hand grip of 3 min to assess the alpha peripheral sympathetic nerve reactivity; the orthostatic or tilt test of 10 min for the study of beta peripheral sympathetic nerve activity; and the deep breathing test for the study of vagal system. The values are expressed as average ± standard deviation.

The comparison of the results between TLE patients with right hippocampal sclerosis and TLE subjects with left hippocampal sclerosis was carried out by means of Student's *t*-test for paired samples. A value *p* < 0.05 was considered significant.

## 4. Results

### 4.1. Demographic and Clinical Features

Thirty patients were recruited in the Department of Clinical Neurophysiology and then addressed to the Unit of Cardiology A, Ibn Sina University Hospital, for performing cardiovascular autonomic test.

A clinical diagnosis and detailed medical history were carried out with special emphasis on types of AEDs taken, interictal EEG recordings, and cranial and hippocampal MRI. The epilepsy type was classified according to the recommendations of the International League Against Epilepsy (ILAE) [23–25].

The interictal EEG registration including T1 and T2 electrodes (International 10-20 system) showed interictal epileptiform discharges for all studied subjects, with 19 patients having left-sided temporal focal spikes and slow waves on the left in the EEG and 11 patients who had right-sided temporal focal spikes and slow waves on the right in the EEG.

The results of analysis on MRI in TLE patients showed 19 patients with left- and 11 patients with right-sided hippocampal sclerosis. Most of patients were under polytherapy with varying combination of two or three antiepileptic drugs, such as sodium valproate (VPA), carbamazepine (CBZ), lamotrigine (LTG), clobazam (CLB), clonazepam (CZP), and phenobarbital (PB). However, only five patients were taking carbamazepine (CBZ) and clobazam (CLB) as monotherapy.

As can be seen from Table 1, there is no significant difference between subjects with left hippocampal sclerosis and patients with right hippocampal sclerosis with respect to age, age of onset, and duration of epilepsy distribution (*p* > 0.05).

Mean basal heart rate (HR) as well as diastolic blood pressure (DBP) did not differ significantly between the two groups. However, the mean basal systolic blood pressure (SBP) differs significantly between patients with left hippocampal sclerosis and patients with right hippocampal sclerosis.

### 4.2. Basal Heart Rate and Blood Pressure

Based on the measurement of basal HR variability in the two groups (TLE with left HS and TLE with right HS) at resting, patients are considered bradycardic if their basal HR is less than 60 beats per minute or tachycardic if their basal HR is greater than or equal to 85 beats per minute.

**Figure 1:**
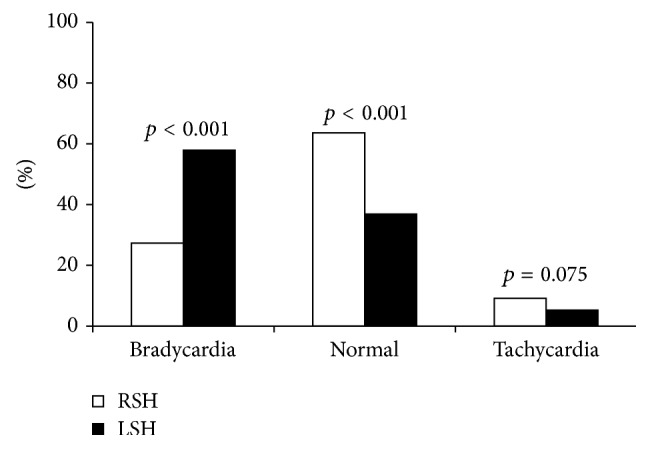
Comparison of basal heart rate in TLE with left and right hippocampal sclerosis.

The occurrence of bradycardia is significantly higher (*p* < 0.001) in TLE with left HS (57.9%) than TLE with right HS (27.27%) (Figure 1), while the prevalence of tachycardia was lower in TLE with left HS (5.2%) with respect to TLE with right HS (9.1%), without any significant difference (*p* = 0.075).

The normal values of basal HR are significantly higher (*p* < 0.001) in TLE with right HS (63.63%) compared to TLE with left HS (36.9%) (Figure 1).

### 4.3. Vagal Response

**Figure 2:**
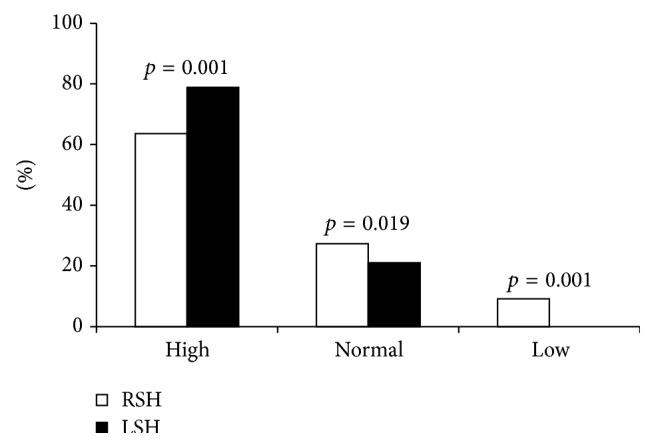
Vagal response on deep breathing test in TLE with right and left hippocampal sclerosis.

The vagal response measured by the deep breathing (DB) test in TLE with right and left hippocampal sclerosis showed a higher parasympathetic activity in TLE patients with left HS (78.9%) than TLE subjects with right HS (63.6%). The difference between the two groups was statistically significant (*p* < 0.001) (Figure 2).

In contrast, a low parasympathetic activity (9.1%) was found in TLE with right HS. However, no case was declared in TLE with left HS. The difference between the two groups was statistically significant (*p* = 0.001). A normal parasympathetic response was higher in TLE with left HS versus right HS. The statistical analysis supports such observation (*p* = 0.004) (Figure 2).

### 4.4. Peripheral Sympathetic Response Alpha

**Figure 3:**
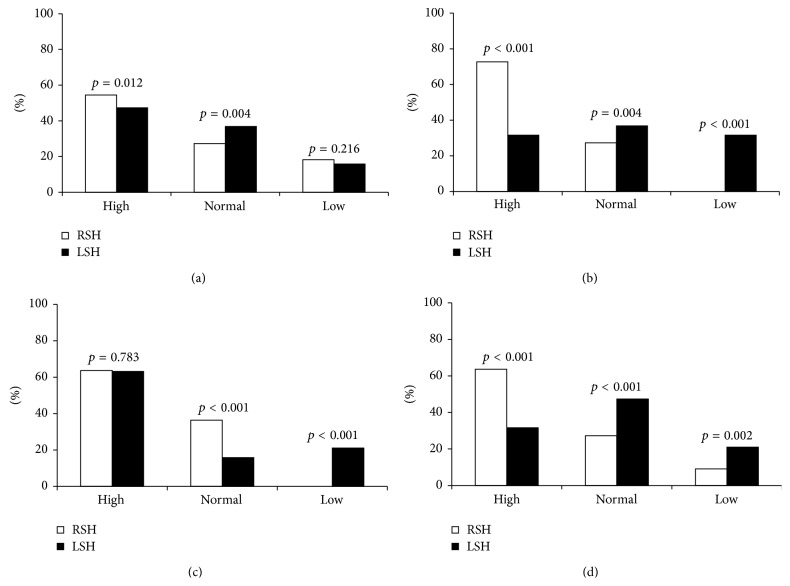
(a) Peripheral sympathetic response alpha (alpha SP) obtained on hand grip test, (b) peripheral sympathetic response beta (beta SP) obtained with orthostatic test, (c) central sympathetic response alpha (alpha SC), and (d) central sympathetic response beta (beta SC) obtained during mental stress.

The peripheral sympathetic response alpha assessed by the hand grip (HG) test in TLE with right hippocampal sclerosis and TLE with a left hippocampal sclerosis showed a significant elevated peripheral sympathetic response alpha in TLE with right HS (54.5%) compared to TLE with left HS (47.36%) (*p* = 0.012, Figure 3(a)), whereas we noted a deficient but not significant (*p* = 0.216) peripheral sympathetic activity alpha in TLE with right HS (18.2%), as compared to TLE with left HS (15.8%). The normal peripheral sympathetic alpha response is slightly higher in TLE with left HS versus TLE with right HS. The statistical analysis supports such observation (*p* = 0.004) (Figure 3(a)).

### 4.5. Peripheral Sympathetic Response Beta

The comparison of the peripheral sympathetic response beta obtained with the orthostatic test (OT) between TLE with right hippocampal sclerosis and TLE with a left hippocampal sclerosis reveals a high peripheral sympathetic beta response in TLE with right HS (72.7%) than TLE with left HS (31.6%). The difference between the two groups was highly significant (*p* < 0.001) (Figure 3(b)). A low peripheral sympathetic beta activity (31.6%) was observed in TLE with left HS. However, there was no noticeable case in TLE with right HS. The difference between the two groups was highly significant (*p* < 0.001). Hence, the normal peripheral sympathetic beta response was found statistically (*p* = 0.004) superior in TLE with left HS (36.8%) with respect to TLE with right HS (27.3%) (Figure 3(b)).

### 4.6. Central Sympathetic Response Alpha

The assessment of the central sympathetic response alpha by the mental stress test, in two groups of TLE, showed a similar high central sympathetic alpha response in TLE with right HS (63.64%) and TLE with left HS (63.16%) (*p* = 0.783) (Figure 3(c)). Deficiency of central sympathetic alpha activity was observed in TLE with left HS (21.05%). However, no declared case was observed in TLE with right HS. The difference between the two groups was highly significant (*p* < 0.001). Normal response of the central sympathetic alpha system is significantly higher (*p* < 0.001) in TLE with left HS (36.8%) compared to TLE with right HS (27.3%) (Figure 3(c)).

### 4.7. Central Sympathetic Response Beta

The central sympathetic response beta evaluated by the mental stress test, in two groups of TLE, showed a highly significant (*p* < 0.001) elevated central sympathetic beta response in TLE with right HS (63.63%) as compared to TLE with left HS (31.59%) (Figure 3(d)). The same observation concerns the deficiency of central sympathetic beta activity, which was significantly important (*p* = 0.002) in TLE with left HS (21.05%) with respect to TLE with right HS (9.1%), while normal response of the central sympathetic beta system was found to be significantly (*p* < 0.001) higher in TLE with left HS (47.36%) in comparison to TLE with right HS (27.27%) (Figure 3(d)).

## 5. Discussion

Through the present study we brought evidence of an association of hippocampal sclerosis with cerebral lateralization that may cause disturbances in parasympathetic and sympathetic autonomic functions during the interictal period in patients with TLE. According to our finding, we revealed profound changes in HR by means of cardiovascular autonomic tests in two groups of TLE that allowed us to identify cerebral lateralization in cardiac autonomic control. Most patients with left-sided temporal focus associated with left HS showed bradycardia, whereas a tendency to tachycardia has been more observed in patients with right-sided temporal focus associated with right HS. Such observation is still under the significance threshold probably due to the low number of sampled patients.

Such finding could be explained by analyzing the sympathetic and parasympathetic activities of the two groups, showing a highly elevated parasympathetic activity in TLE with left HS compared to TLE with right HS. Moreover, comparison of the sympathetic cardiac response between the two groups demonstrates an increase of peripheral and central sympathetic response alpha and beta in patients with right-sided temporal focus associated with right HS, contrary to those with left-sided temporal focus associated with left HS.

We then suggest a possibility of different contributions between the right and left cerebral hemispheres. The left hemisphere predominantly modulates cardiac parasympathetic tone, by increasing its activity, and consequently provokes bradycardia, while the right hemisphere predominantly modulates cardiac sympathetic tone, by increasing its activity, and accordingly causes tachycardia that fits previous studies [7, 11–13, 25, 26].

In another study of humans, Oppenheimer et al. [8] showed that the electrical stimulation of the insular cortex was responsible for cardiovascular changes. Additional investigation showed that bradycardia was often observed during left insular stimulation, while tachycardia occurred during right insular stimulation [7]. Furthermore, neuroimaging studies, animal studies, and clinical observations propose a hemispheric lateralization of parasympathetic or sympathetic cardiovascular control [7, 11, 12, 27–31]. However, results of diverse investigations are contradictory.

Many studies have demonstrated that the right hemisphere dominates sympathetic cardiovascular activity [7, 11, 12, 27–31]. Critchley et al. [31] reported an association between arousal-induced sympathetically mediated skin conductance responses and activity predominantly localized in the right orbitofrontal cortex and the right anterior insula. A rise in regional blood flow in the right insular over stressful tasks associated with sympathetic activation using positron emission tomography was observed in healthy persons. Though, left insular activation concurred with BP and HR diminution during nonstrenuous tasks [29].

Contrary to the findings in healthy subjects, investigations in patients are more equivocal. Oppenheimer et al. [7] reported on subjects with complex partial seizures right-sided dominance of sympathetic BP and HR modulation by intraoperative electrical insular stimulation. With seizure subjects incurring presurgical assessment, Zamrini et al. [12] noticed a hastening of HR after left hemispheric inactivation during intracarotid amobarbital procedure. Likewise, Yoon et al. [11] stated improved sympathetic HR modulation during left-sided intracarotid amobarbital procedure and inferred that there is right hemispheric lateralization of cardiac sympathetic control. Conversely, other authors observed a decrease of parasympathetic HR modulation after right hemispheric stroke [27, 32]. Robinson et al. [33] stated a change in sympathovagal balance with elevated sympathetic activity after right hemispheric lesions in acute stroke patients. This result sustains the assumption of parasympathetic cardiac modulation in the right hemisphere.

Ansakorpi et al. [34] recently showed by investigating the role of hippocampal sclerosis directly in patients with TLE that cardiovascular autonomic dysfunction tended to be greater in patients with hippocampal sclerosis.

Moreover, the cardioregulatory function is a continuously functioning network widely in the inner temporal lobes with various ascending and descending connections mediated by various biochemical neurotransmitters to other areas of the brain [10, 35]. The experimental and clinical data showed the important role of the insular and limbic structures, such as amygdala, in cerebrogenic cardiovascular disturbances in sudden unexpected death in epilepsy patients (SUDEP) [36].

The anatomic or functional infractions such as a neuronal loss and excessive inhibition may cause interictal hypometabolism. Strong projections from limbic to hypothalamic areas and link of autonomic brainstem nuclei to hippocampus suggest that alterations in hippocampal anatomy and/or physiology may provoke disturbances in the autonomic function in patients with temporal epilepsy [37].

In addition to epilepsy itself, the antiepileptic drugs may alter the cardiovascular system as well as the centrally mediated cardiovascular control system function. An interaction between cardiovascular disturbances and AEDs may also contribute as a mechanism of sudden unexpected death in epileptic patients (SUDEP) [38, 39].

## 6. Conclusion

The present study is of a crucial importance, since it brought evidence of a central implication in the sudden unexpected death in temporal epileptic patients. Thus, intracranial disorders associated with hemispheric lateralization, such as hippocampal lesions, are strongly linked to disturbed cardiac features causing bradycardia or tachycardia. Nevertheless, several hypotheses related to possible factors affecting interictal autonomic dysfunction in patients with TLE, factors other than the structural abnormality of hippocampal sclerosis, such as refractoriness and chronic nature of epilepsy, epilepsy itself, duration of epilepsy, drug effects, infrequent seizures, were suggested to be related. Thus, further studies are needed to investigate factors affecting autonomic dysfunction in order to understand and prevent SUDEP.

## Figures and Tables

**Table 1:** Demographics and resting autonomic parameters of thirty temporal lobe epilepsy patients with hippocampal sclerosis.

Parameters	TLE with left HS (*N* = 19)	TLE with right HS (*N* = 11)	All patients (*N* = 30)	*p* values
	(mean ± SD)	(mean ± SD)	(mean ± SD)	
Age (years)	32.47 ± 9.90	32 ± 11.71	32.3 ± 10.40	0.788
Gender (F/M)	14/5	4/7	18/12	0.044
Age of onset of epilepsy (years)	7.37 ± 4.6	8.09 ± 6.8	7.63 ± 5.4	0.682
Duration of illness (years)	25.11 ± 11.75	23.91 ± 11.07	24.67 ± 11.32	0.503
HR (bpm)	63.63 ± 6.78	67.09 ± 9.80	64.90 ± 8.04	0.101
SBP (mm Hg)	109.82 ± 10.27	115.4 ± 11.13	111.87 ±10.76	0.027
DBP (mm Hg)	68.45 ± 12.11	73 ± 7.74	70 ± 10.78	0.057
Frequency seizures	16 ± 13	8 ± 5	12 ± 9	0.003

Values expressed as mean ± SD. *p* is significant if < 0.05. Student's *t*-test.

SBP: systolic blood pressure; DBP: diastolic blood pressure; HR: heart rate; HS: hippocampal sclerosis; F: female; M: male.
